# The Effect of Intravitreal Ranibizumab Injection on Retinal Nerve Fiber Layer Thickness and Optic Disc Parameters

**DOI:** 10.3390/jcm15020449

**Published:** 2026-01-07

**Authors:** Gülin Tuğba Ongun, Ramazan Yağcı

**Affiliations:** 1 Department of Ophthalmology, Antalya City Hospital, Antalya 07230, Turkey; 2 Department of Ophthalmology, Isparta Private Hospital, Isparta 32040, Turkey

**Keywords:** retinal nerve fiber thickness, ranibizumab, age related macular neovascularization

## Abstract

**Objectives:** To evaluate the effects of intravitreal ranibizumab on retinal nerve fiber layer (RNFL) thickness and optic disc parameters in patients treated for exudative age-related macular degeneration (AMD), diabetic macular edema (DME), and retinal vein occlusion (RVO). **Methods:** This retrospective study analyzed the clinical records of 60 patients who received intravitreal ranibizumab injections for macular edema secondary to AMD, DME, or RVO between October 2014 and January 2016. All patients received intravitreal ranibizumab at a dose of 0.5 mg. Best-corrected visual acuity (BCVA) and intraocular pressure (IOP) were recorded at baseline and during follow-up. RNFL thickness and optic disc parameters were assessed using optical coherence tomography (OCT) and Heidelberg Retina Tomograph III (HRT-3). Measurements were obtained before treatment and at 1 week, 1 month, 3 months, and 6 months after injection. Comparisons were performed within and between disease groups. **Results:** Of the 60 patients, 31 (51.7%) had DME, 20 (33.3%) had AMD, and 9 (15.0%) had RVO. Best-corrected visual acuity improved significantly during the follow-up period. Mean RNFL thickness measured by OCT showed a significant reduction in the DME and RVO groups (*p* = 0.0001 and *p* = 0.043, respectively). In contrast, no significant changes in RNFL thickness were detected using HRT-3, and no consistent alterations in other optic disc parameters were observed. Changes in optic disc parameters varied among disease groups. **Conclusions:** Intravitreal ranibizumab treatment was associated with a reduction in mean RNFL thickness measured by OCT in patients with DME and RVO during a six-month follow-up period, whereas no corresponding RNFL thinning was detected using HRT-3 in any disease group. The observed optic disc parameter changes appeared to be disease specific. Given the absence of untreated control eyes and the exclusion of patients with glaucoma, these findings apply only to non-glaucomatous eyes and should not be extrapolated to patients with glaucoma. Further prospective studies with larger cohorts, appropriate control groups, and longer follow-up durations are warranted to clarify the long-term effects of anti-VEGF therapy on the optic nerve.

## 1. Introduction

Angiogenesis plays a critical role in the pathogenesis of diseases such as diabetic maculopathy, age-related macular degeneration (AMD), and retinal vascular occlusion (RVO)-related macular edema [1]. Numerous growth factors involved in angiogenesis have been identified, with vascular endothelial growth factor A (VEGF-A) being the most strongly associated with neovascularization. Ranibizumab is a humanized antibody fragment approved by the FDA (Food and Drug Administration) for intravitreal use targeting all isoforms of VEGF-A [1,2]. Diabetic maculopathy, characterized by foveal edema, ischemia, and exudates, is the leading cause of vision loss, particularly among patients with type 2 diabetes mellitus (DM) [3]. The Diabetic Retinopathy Laser Ranibizumab-Triamcinolone study demonstrated that monthly intravitreal injections of 0.5 mg ranibizumab for three months in patients with foveal edema result in better visual acuity and optical coherence tomography (OCT) outcomes compared to laser treatment alone [4,5].

Glaucoma is considered the most common cause of irreversible visual impairment, whereas AMD ranks as the second leading cause of preventable vision loss [3]. Choroidal neovascularization (CNV) involves the proliferation of new choroidal vessels into the subretinal space. Intravitreal anti-VEGF therapy is widely used in CNV treatment and has been shown to improve visual prognosis [6,7]. In retinal vein occlusion (RVO), macular edema and retinal ischemia are the primary causes of vision loss [8]. Hypoxia-induced upregulation of VEGF in RVO contributes to vascular abnormalities and macular edema, and intravitreal ranibizumab effectively improves visual acuity in these cases [9].

The retinal nerve fiber layer (RNFL) is the innermost layer of the retina, comprising axons of retinal ganglion cells, astrocytes (supporting cells), retinal veins, and extensions of Müller cells [10,11]. This study aims to evaluate RNFL thickness in patients receiving intravitreal ranibizumab injections for macular edema secondary to diabetic retinopathy, AMD, or RVO. Optic disc parameters and RNFL thickness measurements were obtained using Heidelberg Retinal Tomography (HRT-3) and Spectralis OCT during routine examinations. Our objective was to compare the effects of intravitreal ranibizumab injections across these patient groups and to assess time-related changes post-injection. Although longer-term studies with control groups are necessary to fully elucidate the effects of ranibizumab on the RNFL, this study provides a valuable comparative perspective across diverse patient populations.

## 2. Materials and Methods

A power analysis was performed to detect the mean difference between two independent groups. Assuming a medium effect size (Cohen’s d = 0.5), an alpha level of 0.05, and a target power of 0.80 (80%), the analysis indicated that 20 participants per group were required. Accordingly, the study was designed to include a total of 60 participants, meeting the necessary sample size.

This retrospective study included clinical records of 60 patients diagnosed with diabetic macular edema (DME), retinal vein occlusion (RVO), or age-related macular degeneration (AMD) who received intravitreal ranibizumab injections at the Ophthalmology Department of Pamukkale University Faculty of Medicine between October 2014 and January 2016. Exclusion criteria were patients who had received intravitreal injections within the previous six months, prior laser therapy, and ocular diseases affecting the optic disc head such as glaucoma or optic neuritis. Additionally, patients with secondary ocular conditions such as corneal opacity, uveitis, or macular dystrophy were excluded.

Intravitreal ranibizumab was administered at a uniform dose of 0.5 mg/0.05 mL in all disease groups. An initial loading phase consisting of three consecutive monthly injections was followed by a treat-and-extend protocol. At each follow-up visit, patients were evaluated using best-corrected visual acuity and optical coherence tomography. Reinjection was performed in the presence of persistent or recurrent intraretinal or subretinal fluid, increased central retinal thickness, or deterioration in visual acuity attributable to disease activity. When disease stability was achieved, injection intervals were gradually extended by 2–4 weeks at the discretion of the treating physician. Details of the ranibizumab dosing regimen, loading phase, injection protocol, mean number of injections, and follow-up duration for each disease group are summarized in Table 1. Injection intervals were shortened if signs of disease reactivation were observed. Fellow eyes were not included as untreated controls because many patients had bilateral disease, and in cases of unilateral involvement, fellow eyes frequently demonstrated subclinical pathology or prior treatment history, precluding their use as reliable internal controls.

All patients underwent best corrected visual acuity (BCVA) assessment using the Snellen chart, intraocular pressure (IOP) measurement in mmHg with a pneumatic tonometer, biomicroscopic examination, and fundus examination via indirect ophthalmoscopy. Additionally, optic disc parameters were evaluated with Heidelberg Retina Tomograph-3 (HRT-3; Heidelberg Engineering, Dossenheim, Germany), and retinal nerve fiber layer (RNFL) thickness was measured using spectral domain optical coherence tomography (OCT; Zeiss, Jena, Germany). These measurements were recorded before injection and at 1 week, 1 month, 3 months, and 6 months post-injection.

The spectral domain OCT scans tissues at approximately 27,000 A-scans per second, utilizing diode light at a wavelength of 830–840 nm. The axial resolution is 5–6 μm, lateral resolution is 15 μm, and scanning depth is 2.3 mm [12]. Peripapillary RNFL thickness was measured at a 3.4 mm diameter circle in RNFL mode by an experienced OCT operator. The device automatically averages three peripapillary measurements; any scans with boundary detection errors were excluded from analysis. RNFL thickness values were recorded for the superior, inferior, nasal, and temporal quadrants, and overall mean RNFL thickness.

HRT-3 measurements were performed without pupillary dilation. Spherical and astigmatic corrections were applied by an experienced operator prior to imaging. For patients with spherical refractive errors greater than 12 diopters or cylindrical errors exceeding 6 diopters, measurements were taken with appropriate corrective lenses. Quality control was ensured by maintaining a standard deviation below 30 and verifying that the green quality line was above the red reference line on the device’s quality graph. Disc contour lines were generated by marking at least four points around the optic nerve head. Moorfield’s regression analysis was used to assess statistical differences between tomographic data and normal age-matched controls. Both OCT and HRT-3 are non-invasive, radiation-free techniques that do not cause discomfort, require no medication or invasive procedures during measurement [13,14,15,16,17].

### Statistical Analysis

Data analysis was performed using SPSS software version 21 (IBM, Chicago, IL, USA). Continuous variables are expressed as mean ± standard deviation, while categorical variables are presented as counts and percentages. For group comparisons, parametric tests were applied when assumptions were met: Independent Samples *t*-test and Analysis of Variance (ANOVA). If parametric assumptions were not satisfied, non-parametric alternatives such as the Mann–Whitney U test and Kruskal–Wallis test were used. For dependent (paired) group comparisons, the Paired Samples *t*-test was utilized when parametric criteria were fulfilled; otherwise, the Friedman test and Wilcoxon signed-rank test were employed. A *p*-value less than 0.05 was considered statistically significant.

## 3. Results

A total of 60 patients were included in the study, comprising 25 females (41.7%) and 35 males (58.3%). Among them, 31 patients (51.7%) had diabetic macular edema (DME), 20 (33.3%) had age-related macular degeneration (AMD), and 9 (15%) had retinal vein occlusion (RVO). The mean age was 64.3 ± 10.63 years (range, 31–83 years). Patients received between 3 and 6 intravitreal ranibizumab injections. Clinical examinations conducted prior to injection and at 1 week, 1 month, 3 months, and 6 months post-injection were evaluated. The mean best corrected visual acuity (BCVA) improved from 0.28 ± 0.22 at baseline to 0.43 ± 0.3 at 6 months for all patients. Mean intraocular pressure (IOP) was 14.92 ± 3.6 mmHg before treatment and 15.47 ± 2.94 mmHg at 6 months. Statistical comparisons of BCVA and IOP are summarized in Table 2.

No statistically significant differences were observed in cup area, disc area, rim area, cup volume, cup-to-disc area ratio, linear cup-to-disc ratio, mean retinal nerve fiber layer (RNFL) thickness, or RNFL cross-sectional area before and after intravitreal ranibizumab injections in all patients as measured by Heidelberg Retina Tomograph (HRT). These optic disc parameters are detailed in Table 3.

Significant thinning of mean RNFL thickness was observed by optical coherence tomography (OCT) when comparing baseline values to those at 1 month, 3 months, and 6 months post-injection in all patients (*p* = 0.0001). Detailed statistical results are presented in Table 4.

In the AMD subgroup, optic disc parameters measured by HRT before and after treatment are shown in Table 5.

No significant change was found in mean RNFL thickness by OCT (*p* = 0.175). However, a significant reduction was detected in the temporal RNFL thickness at 6 months compared to baseline (*p* = 0.023). Within the DME group, no significant differences were found in HRT variables except for maximum cup depth, mean cup depth, and height variation contour at any time point post-injection. Central foveal thickness and RNFL thickness values measured by OCT in DME patients are presented in Table 6.

For the RVO group, comparison of HRT measurements before injection and at 6 months showed a significant decrease in rim volume and a significant increase in height variation contour (*p* = 0.045 and *p* = 0.02, respectively). Central foveal thickness and RNFL thickness measured by OCT in RVO patients are provided in Table 7.

## 4. Discussion

Anti-VEGF agents require multiple intravitreal injections due to their temporary effects, which increases the risk of ocular complications. These may include transient intraocular pressure (IOP) spikes, frequent IOP fluctuations, and alterations in ocular blood flow that could potentially affect the optic nerve head. Several studies have reported short-term elevations in IOP following intravitreal anti-VEGF injections, and such episodic increases may pose a risk for glaucomatous damage to the optic nerve head [18,19]. Although ranibizumab was generally well tolerated in large clinical trials such as MARINA, ANCHOR, and FOCUS, an increased risk of systemic hemorrhage was reported in control groups, highlighting concerns regarding potential systemic adverse events [20,21,22].

In the present study, no serious ocular or systemic complications—including retinal detachment, vitreous hemorrhage, sudden vision loss, cerebrovascular events, or myocardial infarction—were observed among the 207 intravitreal injections administered to 60 patients. VEGF-A is known to exert neurotrophic and neuroprotective effects on glial cells [23], potentially mediated directly or indirectly through angiogenesis [24,25]. It has therefore been hypothesized that chronic VEGF suppression through repeated anti-VEGF therapy could result in neuronal loss by inhibiting these protective mechanisms. However, multiple experimental studies have suggested that repeated intravitreal anti-VEGF injections are not toxic to the retina [25].

Seth et al. [26] retrospectively evaluated the cup-to-disc (C/D) ratio using fundus photographs in eyes receiving more than two intravitreal injections. In their cohort of 23 eyes with a mean follow-up of nine months, no significant changes in IOP or mean C/D ratio were observed compared with untreated eyes, although one patient required topical antiglaucomatous therapy after a glaucoma diagnosis. In our study, patients with pre-existing glaucoma were excluded to minimize confounding factors related to RNFL loss. Consistent with Seth et al., no significant changes in IOP or C/D ratio measured by Heidelberg Retina Tomograph (HRT) were detected during the six-month follow-up period in any patient group. Nevertheless, glaucomatous eyes may be more susceptible to IOP fluctuations, and this issue warrants further investigation in studies including glaucoma patients and appropriate controls. Similarly, Jayoung Ahn et al. [27] reported no significant differences in peripapillary RNFL thickness or IOP between treated and fellow eyes in patients with exudative AMD.

Previous studies evaluating the effect of ranibizumab on RNFL thickness have yielded conflicting results. Martínez-de-la-Casa et al. [28] reported significant RNFL thinning by OCT after 12 months of ranibizumab treatment in patients with choroidal neovascularization (CNV), whereas El Ashry et al. [29] found no significant thinning after three consecutive injections over a shorter follow-up period. These findings suggest that treatment duration may influence RNFL outcomes. Parlak et al. [30] also reported no statistically significant difference in RNFL thickness between treated CNV eyes and controls during a 12-month treat-and-extend regimen, although RNFL thinning compared with baseline was observed in both groups.

In our study, among 20 patients with CNV treated with ranibizumab, no significant reduction in mean RNFL thickness was detected by OCT after six months, except in the temporal quadrant. However, a significant decrease in cup-to-disc surface mean and an increase in cup volume were observed on HRT measurements. These changes were unique to the CNV group and may indicate subtle optic disc alterations not fully captured by RNFL thickness alone. Further studies with larger sample sizes and appropriate control groups are required to clarify the clinical significance of these findings.

Shin et al. [31] reported no significant differences in RNFL thickness between treated and untreated eyes in patients with DME, AMD, and RVO over a 12-month period. RNFL thinning was observed in both treated and untreated DME and RVO eyes but not in AMD, suggesting that ischemic mechanisms inherent to these diseases, rather than anti-VEGF toxicity, may be responsible for RNFL loss. Our findings are consistent with these results, as significant RNFL thinning was observed in the DME and RVO groups by OCT, whereas no significant changes were detected by HRT.

Previous studies in patients with diabetes mellitus have reported no significant changes in optic disc size, shape, or neuroretinal rim area, although ophthalmoscopic evaluation of RNFL remains challenging due to optic disc pallor [32,33]. Takahashi and Chihara [34] described RNFL defects in diabetic patients without an associated increase in the C/D ratio, suggesting that diabetes-related RNFL damage may occur independently of optic disc morphology. In our study, no significant changes in mean RNFL thickness or C/D ratio were observed in DME patients treated with ranibizumab. However, significant RNFL thinning was detected in specific quadrants by OCT, accompanied by an increase in height variation contour on HRT. Notably, RNFL thinning was more pronounced in DME patients than in AMD and RVO groups.

To more clearly delineate the effects of anti-VEGF therapy from the natural disease course, comparison between treated and untreated DME and RVO patients would be ideal; however, withholding treatment poses significant ethical challenges given its established role in the management of vision-threatening macular edema.

Overall, changes in optic disc parameters—such as thinning of cup surface mean and increased cup volume in AMD patients, decreased rim volume in RVO patients, and increased height variation contour in DME and RVO patients—suggest that anti-VEGF therapy may influence retinal nerve structures beyond RNFL thickness alone. No significant IOP changes or IOP-related RNFL alterations were observed during the six-month follow-up period; however, chronic IOP fluctuations remain a potential risk factor for glaucomatous damage.

The absence of untreated control eyes represents an important limitation of the present study, as it restricts the ability to conclusively attribute the observed RNFL and optic disc changes to anti-VEGF therapy rather than to the natural course of the underlying retinal diseases. Nevertheless, available natural-history data indicate that RNFL thinning may occur independently of treatment, particularly in ischemic conditions such as DME and RVO. The pattern observed in our study—significant RNFL thinning in DME and RVO but not in AMD—is consistent with these observations. In addition, the exclusion of patients with pre-existing glaucoma limits the generalizability of our findings, as glaucomatous eyes may be more susceptible to optic nerve damage related to intraocular pressure fluctuations or potential anti-VEGF-associated effects. Ethical considerations limit the feasibility of withholding anti-VEGF therapy and of including untreated high-risk subgroups such as glaucoma patients; therefore, future prospective studies with larger cohorts, longer follow-up durations, and appropriate control or natural-history comparator groups, including glaucoma patients as a separate subgroup, are warranted to better clarify the relationship between anti-VEGF therapy and optic nerve changes. Accordingly, the conclusions of the present study apply only to non-glaucomatous eyes and should not be extrapolated to patients with glaucoma. Another limitation of this study is the lack of fellow-eye comparisons in patients with unilateral disease, which may have otherwise provided an internal control minimizing systemic confounders.

The discrepancy observed between significant RNFL thinning detected by OCT and the absence of corresponding RNFL changes on HRT-3 may be explained by fundamental technical and methodological differences between the two imaging modalities. OCT provides high-resolution, layer-specific cross-sectional measurements of the peripapillary RNFL and is particularly sensitive to localized or early structural changes. In contrast, HRT-3 is a confocal scanning laser ophthalmoscopy-based technique that primarily evaluates optic disc topography and derives RNFL-related parameters indirectly, which may reduce its sensitivity to subtle or focal RNFL thinning.

Additionally, differences in segmentation algorithms, reference planes, scan geometry, and susceptibility to operator-dependent contour line placement may further contribute to discordant findings between OCT and HRT measurements. Similar discrepancies between OCT- and HRT-derived RNFL assessments have been reported in previous studies, particularly in early or non-glaucomatous optic nerve changes, supporting the notion that these modalities may capture different aspects of optic nerve head and RNFL integrity rather than providing interchangeable measurements [35]. Baseline correlations between OCT- and HRT-3-derived RNFL measurements were not evaluated, which represents an additional limitation of the study.

Another important limitation of this study is the relatively short follow-up duration of six months. Although this period is sufficient to detect early or short-term structural changes in RNFL and optic disc parameters, it is inadequate to assess potential chronic or cumulative effects of long-term anti-VEGF therapy, which is frequently administered over several years in routine clinical practice. Therefore, the findings of the present study should be interpreted as reflecting short-term outcomes only. Longer-term prospective studies with extended follow-up periods are required to determine whether the observed changes remain stable, progress, or translate into clinically meaningful optic nerve damage over time. An additional limitation of this study is the relatively small sample size of the RVO subgroup (n = 9), which is below the number required by the a priori power analysis and may limit the statistical power to detect subtle RNFL or optic disc changes in this group.

## 5. Conclusions

As far as we know, there is no information in the literature regarding changes in retinal nerve fiber layer (RNFL) thickness and optic disc parameters evaluated with Heidelberg Retina Tomograph (HRT) in patients receiving multiple intravitreal ranibizumab treatments. To this end, studies with larger patient samples, control groups, and longer follow-up periods are warranted. In conclusion, intravitreal ranibizumab treatments administered to patients with age-related macular degeneration (AMD), diabetic macular edema (DME), and retinal vein occlusion (RVO) did not cause significant thinning in mean RNFL thickness as measured by HRT. The decrease in RNFL thickness observed with optical coherence tomography (OCT) in DME and RVO patients may be attributed to retinal ischemia rather than anti-VEGF treatment. Accordingly, the conclusions of this study are limited to a six-month follow-up period and cannot be extrapolated to long-term anti-VEGF treatment.

## Figures and Tables

**Table 1:** Ranibizumab Dosing and Injection Protocols by Disease Group.

Parameter	DME	AMD	RVO
Ranibizumab dose	0.5 mg	0.5 mg	0.5 mg
Loading phase	Yes (3 injections)	Yes (3 injections)	Yes (3 injections)
Injection protocol	Treat-and-extend	Treat-and-extend	Treat-and-extend
Mean number of injections	7	6	6
Mean follow-up duration (months)	6	6	6

DME: diabetic macular edema; AMD: age-related macular degeneration; RVO: retinal vein occlusion.

**Table 2:** Values of BCVA and IOP before and after intravitreal Ranibizumab injection.

		Pre-Injection	Post-Injection 6th Month	*p* Value	Test Value
**BCVA** **(SNELLEN)**	**AMD**	0.28 ± 0.24	0.39 ± 0.32	**0.009**	**T = 2.61**
	**DME**	0.29 ± 0.20	0.46 ± 0.30	**0.0001**	**T = 3.04**
	**RVO**	0.22 ± 0.22	0.39 ± 0.30	**0.043**	**T = 1.44**
	**P1**	0.676	0.604		
**IOP** **(mmHg)**	**AMD**	13.85 ± 3.56	15.25 ± 3.57	0.177	T = 0.36
	**DME**	15.42 ± 3.58	15.55 ± 2.59	0.79	T = 0.15
	**RVO**	15.56 ± 3.68	15.67 ± 2.87	0.912	T = 0.10
	**P1**	0.272	0.593		

BCVA: best-corrected visual acuity; IOP: intraocular pressure; AMD: age-related macular degeneration; DME: diabetic macular edema; RVO: retinal vein occlusion. Values are presented as mean ± standard deviation. A *p* value < 0.05 was considered statistically significant. Paired *t*-test was used for within-group comparisons.

**Table 3:** Values of optical disk parameters before and after intravitreal Ranibizumab injection in HRT measurements.

	Pre-Injection	1st Week	1st Month	3rd Month	6th Month		
**Patients (n = 60** **)**	(Mean ± SD)	(Mean ± SD)	(Mean ± SD)	(Mean ± SD)	(Mean ± SD)	***p* value**	**Test value**
**Disc Area**	2.23 ± 0.4	2.22 ± 0.4	2.22 ± 0.4	2.22 ± 0.4	2.23 ± 0.4	0.817	F = 0.3
**Cup Area**	0.3 ± 0.2	0.32 ± 0.3	0.32 ± 0.3	0.32 ± 0.3	0.34 ± 0.3	0.223	F = 0.8
**Rim Area**	1.93 ± 0.4	1.9 ± 0.4	1.9 ± 0.4	1.91 ± 0.4	1.91 ± 0.4	0.452	F = 1.1
**Cup Volume**	0.06 ± 0.07	0.06 ± 0.08	0.06 ± 0.07	0.06 ± 0.07	0.08 ± 0.1	0.128	F = 0.75
**Rim Volume**	0.58 ± 0.3	0.64 ± 0.3	0.62 ± 0.3	0.59 ± 0.2	0.62 ± 0.3	**0.015**	**F = 6.7**
**Cup/Disc Ratio (c/d)**	0.13 ± 0.1	0.14 ± 0.12	0.15 ± 0.1	0.15 ± 0.1	0.14 ± 0.12	0.443	F = 1.01
**Linear c/d**	0.32 ± 0.1	0.32 ± 0.2	0.33 ± 0.1	0.33 ± 0.2	0.33 ± 0.2	0.715	F = 0.27
**Mean Cup Depth**	0.16 ± 0.1	0.16 ± 0.09	0.16 ± 0.09	0.16 ± 0.09	0.16 ± 0.09	**0.005**	**F = 8.2**
**Max. Cup Depth**	0.46 ± 0.2	0.48 ± 0.2	0.48 ± 0.2	0.46 ± 0.2	0.47 ± 0.2	**0.005**	**F = 8.8**
**CSM**	0.22 ± 0.07	0.21 ± 0.07	0.22 ± 0.07	0.21 ± 0.06	0.2 ± 0.07	**0.027**	**F = 5.8**
**HVC**	0.39 ± 0.1	0.44 ± 0.1	0.43 ± 0.1	0.41 ± 0.1	0.43 ± 0.1	**0.001**	**F = 9.7**
**Mean RNFL Thickness**	0.25 ± 0.08	0.26 ± 0.09	0.26 ± 0.09	0.24 ± 0.09	0.26 ± 0.1	0.145	F = 0.68
**RNFL CSA**	1.31 ± 0.5	1.37 ± 0.5	1.38 ± 0.5	1.27 ± 0.4	1.38 ± 0.6	0.161	F = 0.49

HRT: Heidelberg retinal tomography; SD: standard deviation; CSM: cup shape measure; HVC: height variation contour; RNFL: retinal nerve fiber layer; CSA: cross-sectional area. One-way ANOVA (*F* test) was used for statistical analysis. A *p* value < 0.05 was considered statistically significant.

**Table 4:** RNFL thickness measurements with OCT.

	Pre-Injection	1st Week	1st Month	3rd Month	6th Month		
**Patients (n = 60)**	(Mean ± SD)	(Mean ± SD)	(Mean ± SD)	(Mean ± SD)	(Mean ± SD)	***p* value**	**Test value**
**Mean RNFL Thickness (µm)**	116.3 ± 28	113.83 ± 27	109.47 ± 23	108.08 ± 22	108.4 ± 23	**0.0001**	**F = 12.1**
**Superior Quadrant RNFL (µm)**	259.78 ± 65	268.67 ± 78	263.9 ± 64	257.53 ± 59	256.93 ± 56	0.213	F = 3.2
**İnferior Quadrant RNFL (µm)**	283.17 ± 51	276.62 ± 50	272.25 ± 47	270.8 ± 47	272.25 ± 48	**0.001**	**F = 10.6**
**Nasal Quadrant RNFL (µm)**	97.72 ± 40	93.48 ± 33	87.88 ± 29	84.53 ± 28	84.42 ± 30	**0.001**	**F = 11.3**
**Temporal Quadrant RNFL (µm)**	92.17 ± 34	88.27 ± 30	85.93 ± 28	88.28 ± 29	86.9 ± 29	**0.0001**	**F = 13.7**

RNFL: retinal nerve fiber layer; OCT: optical coherence tomography; SD: standard deviation. One-way ANOVA (*F* test) was used for statistical analysis. A *p* value < 0.05 was considered statistically significant.

**Table 5:** Values of optic disc parameters, measured with HRT, in the AMD patient group.

	Pre-Injection	1st Week	1st Month	3rd Month	6th Month		
**AMD (n = 20)**	(Mean ± SD)	(Mean ± SD)	(Mean ± SD)	(Mean ± SD)	(Mean ± SD)	***p* value**	**Test value**
**Disc area**	2.21 ± 0.3	2.22 ± 0.4	2.21 ± 0.4	2.21 ± 0.3	2.23 ± 0.4	0.406	F = 2.7
**Cup area**	0.29 ± 0.2	0.3 ± 0.3	0.3 ± 0.3	0.33 ± 0.3	0.33 ± 0.3	0.117	F = 1.01
**Rim area**	1.92 ± 0.3	1.9 ± 0.3	1.9 ± 0.2	1.88 ± 0.3	1.9 ± 0.2	0.417	F = 2.6
**Cup volume**	0.05 ± 0.07	0.06 ± 0.09	0.06 ± 0.09	0.06 ± 0.08	0.1 ± 0.2	**0.032**	**F = 6.90**
**Rim volume**	0.59 ± 0.2	0.61 ± 0.17	0.64 ± 0.2	0.58 ± 0.2	0.59 ± 0.3	0.456	F = 3.1
**Cup/disc ratio (c/d)**	0.12 ± 0.09	0.13 ± 0.1	0.13 ± 0.2	0.14 ± 0.1	0.14 ± 0.1	0.147	F = 1.3
**CSM**	0.22 ± 0.05	0.21 ± 0.05	0.22 ± 0.04	0.2 ± 0.04	0.2 ± 0.06	**0.018**	**F = 7.23**

HRT: Heidelberg retinal tomography; AMD: age-related macular degeneration; CSM: cup shape measure; SD: standard deviation. One-way ANOVA (*F* test) was used for statistical analysis. A *p* value < 0.05 was considered statistically significant.

**Table 6:** Central foveal thickness and RNFL thickness values in OCT measurements in the DME patient group.

	Pre-Injection	1st Week	1st Month	3rd Month	6th Month		
**DME group (n = 31)**	(Mean ± SD)	(Mean ± SD)	(Mean ± SD)	(Mean ± SD)	(Mean ± SD)	***p* value**	**Test value**
**Central foveal thickness** **(µm)**	498.71 ± 132	393.71 ± 92	366.81 ± 90	334.9 ± 81	325.29 ±90	**0.0001**	**F = 16.8**
**Mean RNFL thickness (µm)**	121.74 ± 31	118.42 ± 29	115.26 ± 27	114.35 ± 26	114.65 ± 27	**0.0001**	**F = 18.2**
**Superior quadrant RNFL thickness** **(µm)**	277.74 ± 67	276.13 ± 67	276.35 ± 62	274.81 ± 63	273.81 ± 58	0.449	F = 2.11
**İnferior quadrant RNFL thickness** **(µm)**	296.06 ± 54	289.55 ± 53	283.9 ± 52	282.42 ± 44	280.65 ± 49	**0.008**	**F = 10.9**
**Nazal quadrant RNFL thickness** **(µm)**	102.19 ± 42	97.65 ± 35	94.94 ± 36	91 ± 36	92.23 ± 37	**0.003**	**F = 10.1**
**Temporal quadrant** **RNFL thickness** **(µm)**	97.74 ± 40	95.23 ± 38	91.77 ± 35	95.16 ± 35	93 ± 34	**0.0001**	**F = 14.6**

RNFL: retinal nerve fiber layer; OCT: optical coherence tomography; DME: diabetic macular edema; SD: standard deviation. One-way ANOVA (*F* test) was used for statistical analysis. A *p* value < 0.05 was considered statistically significant.

**Table 7:** Central foveal thickness and RNFL thickness values in the RVO patient group measured with OCT.

	Pre-Injection	1st Week	1st Month	3rd Month	6th Month		
**RVO (n = 9)**	(Mean ± SD)	(Mean ± SD)	(Mean ± SD)	(Mean ± SD)	(Mean ± SD)	***p* value**	**Test value**
**Central foveal thickness (µm** **)**	483.89 ± 97	377.44 ± 99	308 ± 68	321.89 ± 87	290.67 ± 62	**0.0001**	**F = 17.9**
**Mean RNFL thickness (µm)**	112.44 ± 3	106.33 ± 23	102.33 ± 22	102 ± 19	106.33 ± 26	**0.043**	**F = 11.1**
**Superior quadrant RNFL thickness (µm)**	246 ± 80	243.67 ± 62	246.56 ± 62	246.44 ± 74	247.22 ± 63	0.413	F = 4.1
**İnferior quadrant RNFL thickness (µm)**	272 ± 70	262.67 ± 70	252.44 ± 55	254.89 ± 62	265.78 ± 73	0.23	F = 2.9
**Nazal quadrant RNFL thickness (µm)**	92.33 ± 28	87.56 ± 19	85 ± 20	83.67 ± 20	86.89 ± 24	0.146	F = 1.7
**Temporal quadrant RNFL thickness (µm)**	93.33 ± 41	83.11 ± 26	79.11 ± 23	81.67 ± 23	83.89 ± 30	0.473	F = 3.8

Values are presented as mean ± standard deviation. RNFL: retinal nerve fiber layer. Repeated-measures ANOVA was used for comparisons across time points. A *p* value < 0.05 was considered statistically significant.

## Data Availability

The data presented in this study are available on request from the corresponding author. The data are not publicly available due to privacy and ethical restrictions.
